# Characterization of B cells in lupus erythematosus skin biopsies in the context of different immune cell infiltration patterns

**DOI:** 10.3389/fmed.2022.1037408

**Published:** 2022-11-10

**Authors:** Luka de Vos, Tugce Guel, Dennis Niebel, Sandra Bald, Adrian ter Steege, Thomas Bieber, Joerg Wenzel

**Affiliations:** ^1^Department of Dermatology and Allergy, University Hospital Bonn, Bonn, Germany; ^2^Department of Dermatology, University Hospital Regensburg, Regensburg, Germany

**Keywords:** inflammation, BLyS, BAFF receptor (BAFF-R), MXA, pDC, interferon, B cell, cutaneous lupus erythematosus

## Abstract

Cutaneous lesions in lupus erythematosus (LE) subtypes are heterogenous. In line with the heterogeneity of the clinical presentation, the underlying lesional inflammation in LE skin samples is defined by different immune cell infiltrates. Pathophysiologically, lesional inflammation is driven by autoreactive cytotoxic T cells, targeting keratinocytes; plasmacytoid dendritic cells (pDCs), producing large amounts of interferon (IFN); and B cells, whose function in cutaneous LE is still unclear. This study aims to (a) classify inflammatory patterns with regard to the dominating cell type or cytokine expression and (b) investigating the specific role of B cells in LE skin lesions. Therefore, the immunohistological expression of inflammatory surrogates (CD20, CD123, MXA) in skin samples of *n* = 119 LE (subtypes: subacute cutaneous LE, chronic discoid LE, chilblain LE, LE tumidus, other LE) and *n* = 17 patients with inflammatory skin diseases (atopic dermatitis, psoriasis) were assessed. Samples were classified with regard to inflammatory groups. In addition multiplex-immunohistochemical analyses of *n* = 17 LE skin samples focusing on lesional B cells were conducted. In this study, we show that cutaneous lesions present with eight different inflammatory groups dominated by B cells, pDCs, a strong IFN expression, or overlapping patterns. Altogether, LE subtypes show heterogenous infiltration regardless of LE subtype, certain subtypes display a preference for infiltration groups. Furthermore, lesional B cells either form diffuse infiltrates or pseudofollicular structures, wherein they show antigen-presenting and T cell-activating properties. Altogether, in the light of emerging targeted therapeutic options, we suggest histological assessment in regard to B-cell or pDC preponderance to allow tailored treatment decisions.

## Introduction

Lupus erythematosus (LE) is a chronic inflammatory disease with potential for systemic or cutaneous involvement only. LE can be accompanied by detectable autoantibodies in the serum, e.g., antinuclear antibodies (ANA) and Ro/SSA antibodies; nevertheless, not all cutaneous LE (CLE) patients harbor circulating antibodies (1, 2). Histologically, CLE presents with an interface dermatitis, an epidermotropic cytotoxic lymphocytic infiltrate at the dermo-epithelial junction, a perivascular and periadnexial lymphocytic infiltrate, as well as overexpression of interferon (IFN) – regulated cytokines (3, 4). The inflammatory infiltrate consists of three main cell types: (1) autoreactive cytotoxic T cells which target lesional keratinocytes; (2) plasmacytoid dendritic cells (pDCs) that produce large amounts of type I interferons within the skin lesions to drive lesional inflammation; (3) skin-associated B cells, whose role in modulation of LE-specific cutaneous immune response is still unclear (5–8). In principle, B cells could have different functions in the lesional autoimmune reaction. B cells could function as autoantibody-producing or as antigen-presenting cells, cytokine producers or as co-stimulatory T cell-activating cells within the lesion (7, 9, 10). It has been shown that skin-resident B cells have the capacity to express high levels of MHC-II and costimulatory molecules (CD80/86), an indication for a function as T cell activators (7, 9, 10).

Currently, the research focus has been drawn emergingly to the B cell infiltrate in CLE. B cell infiltration varies in the context of CLE subtypes (11). B lymphocyte stimulator [BLyS, also known as B cell activating factor (BAFF)] is a membrane bound or soluble factor involved in B cell maturation and B cell survival (12). BLyS is a survival factor for B cells that is crucial for B cell maturation as it supports the survival of immature transitional B cell type II (T2 B Cell) and possibly mature B cells (12). Among other cells, BLyS is expressed by keratinocytes (13). In a previous study, our group analyzed epidermal expression to detect preferential expression in the lower epidermis with the strongest expression in the inflammatory infiltrate (“interface dermatitis”) (13). The associated BAFF-Receptor (BAFF-R) is expressed by the lymphocytic infiltrate (12, 13). BAFF/BlyS is IFN-regulated, produced by keratinocytes in CLE after stimulation of PRRs (13). Therefore, it may play an important role in the intersection between innate and adaptive immune system in CLE (13). A monoclonal antibody targeting BLyS (Belimumab) is approved for treatment of highly active, autoantibody-positive systemic lupus erythematosus (SLE) despite standard treatment (14). Interestingly, Belimumab has also shown efficacy in CLE patients without elevated autoantibody serum levels (1, 2, 11), indicating an additional role for B cells apart from antibody production.

Altogether, we hypothesize that lesional inflammation presents with heterogeneous patterns driven by pCDs or B cells. Since the specific role of B cells in the autoimmune reaction in CLE is still unclear, we aim to characterize lesional B cells in lupus skin samples. With emerging treatment options, further classification based on the immune infiltration pattern could optimize the individual therapy management of CLE patients. Therefore, this study strives for a comprehensive characterization of the inflammatory subgroups regardless of lupus subtype.

## Materials and methods

### Patient samples and ethics

In total, *n* = 136 skin biopsies including CLE and systemic LE (SLE), and other inflammatory skin diseases [psoriasis (PSO), atopic dermatitis (AD)] were analyzed. This includes *n* = 84 biopsies from patients with CLE from Biobank Bonn and additionally *n* = 35 lupus biopsies from the ongoing BeliSkin study (GSK-supported ISS, EudraCT Number: 2017-003051-35). Therefore, in total, *n* = 119 lupus samples and *n* = 17 inflammatory controls were included. The diagnoses of all patients (LE and inflammatory controls) were clinico-pathologically confirmed at the University Hospital Bonn. The LE samples were classified with regard to clinical and histopathological findings: systemic LE (*n* = 3), acute- (ACLE) (*n* = 1), subacute LE (SCLE) (*n* = 39), intermittent / lupus erythematosus tumidus (ICLE, LET) (*n* = 15), and chronic, not further classified CLE (*n* = 5), chronic discoid LE (CDLE) (*n* = 45), and chilblain LE (ChLE) (*n* = 11) (15, 16). ACLE, SLE and unclassified CLE cases were considered “other LE.”

Detailed information of the study cohort is seen in Supplementary Table 1.

The study was performed as an in-vitro and ex-vivo study. All patients were treated between 01/2017 and 03/2022 at the University Hospital of Bonn. All biopsies were taken for diagnostic purposes at times of active skin disease. The study was performed in accordance with the Declaration of Helsinki principles. The study was approved the local ethical committee of Bonn (090/04, 153/18AMG-ff).

### Histology

Histological and immunohistochemical analyses were conducted from 4mm skin-punch biopsies taken for diagnostic purposes. All sections were prepared from formalin-fixed and paraffin-embedded skin biopsy samples. Standard hematoxylin & eosin (H&E) stain and periodic acid-Schiff staining was performed. For this study, H&E stained sections were used to semi-quantify the extent and intensity of the histopathological changes. In detail, vacuolar alteration of the basal layer as measure for the extent of interface dermatitis, and abundance of mucin were scored from 0 to 3 (0: absent; 1: scant; 2: plenty; 3: abundant). The distribution of lymphocytic infiltrate (peri-adnexal, peri-vascular), and the intensity of neutrophilic infiltrate were each scored from 0 to 3 (0: no expression; 1: single cell; 2: intermediate; 3 = strong expression of diffuse or clustered cells). Positive cells were counted per x200 high power field as described previously (17). B cells that were arranged in clusters are referred to as pseudofollicularly arranged / pseudofollicular B cell formation (18–20).

### Immunohistochemistry

In addition to the H&E stained slides, immunohistochemical staining was performed. For immunohistochemical staining, biopsies were fixed in 5% formalin solution overnight. We used the ZytoChem Plus AP Polymer System (Mouse/Rabbit) with Zytomed AP-Red Kit following the manufacturer’s protocol (Zytomed Systems GmbH, Berlin, Germany). The following monoclonal antibodies were used: MXA (M143 University Medical Centre Freiburg, Germany, 1 μg/ml), CD3 (DAKO, Jena, Germany, GA503, ready-to-use), CD123, (BD biosciences, New Jersey, USA, 554528, 2.5 μg/ml), CD20 (DAKO, Jena, Germany, GA604, ready-to-use), BAFF-R (antikoerper-online, Aachen, Germany, ABIN207878, 3 μg/ml). All antibodies were diluted with Zytomed Antibody Diluent (ZUC025-100) from Zytomed Systems GmbH, Berlin, Germany.

To characterize the immune infiltrate, the following immunohistochemical stainings were performed: CD20 as surrogate for B cells, CD3 as surrogate for T cells, and CD123 as surrogate for pDCs. Infiltrating immune cells were semi-quantified (with 0: no expression; 1: single cell; 2: intermediate; 3 = strong expression of diffuse or clustered cells) as described above for the neutrophilic granulocytes. As surrogate for the interferon signature, the pan-interferon-marker myxovirus resistance protein A1 (MXA) was used and was semi-quantified (with 0: absent; 1: scant; 2: plenty; 3: abundant). In addition, the expression of B-cell activation markers BAFF-Receptor/BAFF-R was also semi-quantified according to the expression as described before for MXA.

### Multiplex-immunohistochemistry

For subtype classification and characterization of B cells, multiplex-immunohistochemistry was performed on *n* = 17 LE samples. We used Ultivue’s (Cambridge, USA) InSituPlex (ISP) platform technology to characterize marker signatures using a custom developed multiplex assay. Ultivue’s InSituPlex technology is based on the detection of conjugated antibodies by fluorophore tagged probes. Antibodies specific for each target are conjugated to short unique oligonucleotides, referred to as barcodes. For each barcode, a unique complementary oligonucleotide probe tagged with a fluorescent dye was used to label the antibody conjugates. Different fluorophores were associated with each barcode–probe pair to spectrally separate the targets during imaging. The following monoclonal antibodies were used: BAFF-R (Abcam, Cambridge, United Kingdom, EPR 14633), CD20 (Thermofisher, Massachusetts, USA, L26), MHC-II (Abcam, Cambridge, United Kingdom, ab7856 CR3/43), CD80 (Novus, Colorado, USA, 37711), CD86 (CST, Walsall, United Kingdom, E2G8P). Imaging exposure times were set per batch of samples. Positive tissue control and negative reagent controls (isotype antibody control and primary antibody delete) will be included in all multiplex staining runs. More detailed information on Ultivue’s (Cambridge, USA) ISP platform’s technology and illustrations of the method are available (21).

Image analysis was performed by Ultivue (Cambridge, USA) on the multi-fluorescent whole slide image to detect and classify all cells using the HALO platform (Indica Labs, v3.3.2541.424). Manual annotations were used to define the regions of interest for image analysis. A semantic segmentation algorithm was developed for each sample to segment the analysis region into tissue and non-tissue compartments. Within the detected tissue, a nucleus segmentation algorithm was developed using a set of DAPI-based morphological and intensity features. Detected nuclei were expanded to include a 2 μm approximated cytoplasm compartment. Single marker positivity was determined using intensity thresholds in the relevant sub-cellular compartment (nuclear, cytoplasmic and/or membrane). Results of the region detection, cell segmentation and cell classification were visually inspected for accuracy. Image analysis results were accepted if approximately 80% of single marker positive cells are correctly identified according to the visual inspection. The described segmentation and classification strategy was used to generate binary classification of cells per marker. Biological interpretability is further improved by adding expert knowledge about marker combinations or cell localization. Cells can be labeled into “phenotypes” (e.g., “B cell”) giving more accurate and intelligible insights into the biology of the analyzed cell populations. Phenotyping was performed by Ultivue with a python-based custom algorithm. Finally, quantitative readouts were generated for the analyzed tissue areas and included absolute and relative cell counts (cell densities and percentages) and cell intensities.

### Statistical analyses

The IBM SPSS-software was used for data analysis. Groups were compared using Mann-Whitney-U and Kruskal-Wallis-tests, and correlation analyses were conducted via Spearman’s ρ correlation analyses. *P* < 0.05 were considered statistically significant.

## Results

### Characterization of the immune cell infiltrate in lupus skin samples of patients with different lupus erythematosus subtypes

**FIGURE 1:**
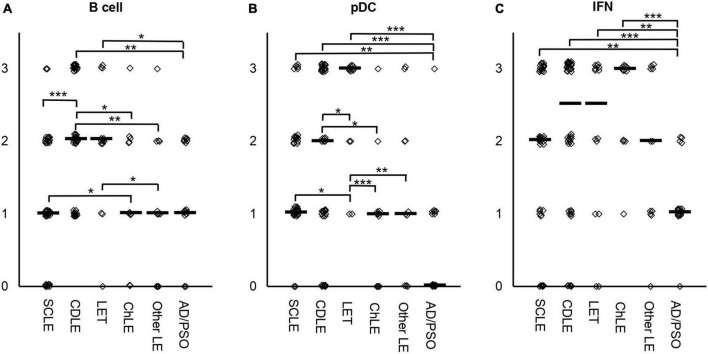
Semi-quantified score for infiltration of B cells **(A)**, pDCs **(B)** or MXA as marker for interferon (IFN) **(C)** in skin samples of *n* = 119 lupus erythematosus (LE) and *n* = 17 inflammatory controls (psoriasis, atopic dermatitis). Immunohistochemical staining was performed to semi-quantify B cells via CD20, pDCs *via* CD123, and IFN expression via MXA staining. CD20 and CD123 expression was scored according to a semi-quantitative system and ranged from 0: no expression; 1: single cell infiltration; 2: intermediate diffuse or clustered infiltration and 3: strong diffuse or clustered infiltration **(A,B)**. MXA expression was semi-quantitatively scored from 0: no expression; 1: scant; 2: plenty to 3: abundant expression **(C)**. The median is highlighted as small bars. Significant differences between groups assessed via Kruskal-Wallis analyses are shown with bars. Significance levels are highlighted as follows: * *p* < 0.05, ** *p* < 0.01, *** *p* < 0.001.

Initially, the lesional CLE immune infiltrates were stratified with regard to high infiltration of pDCs, B cells, and an interferon-signature assessed via CD20, CD123, and MXA expression, respectively. CD20 and CD123 expression was scored according to a semi-quantitative system and ranged from 0: no expression; 1: single cell infiltration; 2: intermediate and 3: strong diffuse or clustered infiltration (Figures 1A,B). Since we observed an overall low cellular infiltration but high MXA expression in a portion of LE skin samples, we also stratified MXA expression as surrogate for IFN-expression. MXA expression was semi-quantitatively scored from 0: no expression; 1: scant; 2: plenty to 3: abundant expression (Figure 1C). Scores > 1 were defined as considered significantly characteristically infiltrated, in summary a total 56% of CLE cases were B cell, 51% pDC infiltrated, and 71% displayed a high interferon-signature.

Infiltration between the LE subtypes varied significantly (Figure 1). CDLE samples harbored highest B cell counts, significantly more compared to SCLE, ChLE, other LE samples and the controls AD/PSO (Figure 1A). B cell infiltration also varied between LET vs. controls and vs. other LE, and SCLE vs. ChLE. Similarly, pDC infiltration varied with highest pDC infiltration in LET, significantly higher compared to all other subtypes (SCLE, CDLE, ChLE, other LE and inflammatory controls) (Figure 1B). pDC infiltration did not show significant differences between SCLE and CDLE. Interestingly, the expression of IFN-surrogate MXA varied between inflammatory controls and all lupus subtypes, but not between lupus subtypes.

In order to further examine infiltration patterns, we correlated the expression of B cells, pDCs, MXA and other inflammatory surrogate markers present in lupus skin lesions. Generally, stronger infiltrated samples were more likely to encompass B cells, pDCs, as well as a high interferon-signature. Accordingly, positive correlations were found for B cells vs. pDCs (Spearman’s ρ = 0.389, *P* = 0.000), vs. neutrophilic infiltrate (Spearman’s ρ = 0.215, *P* = 0.003), vs. MXA scoring (Spearman’s ρ = 0.305, *P* = 0.000), vs. periadnexial (Spearman’s ρ = 0.576, *P* = 0.000), and perivascular lymphocytic infiltrate (Spearman’s ρ = 0.475, *P* = 0.000), vacuolar alteration of basal layer (Spearman’s ρ = 0.151, *P* = 0.042), and abundance of mucin (Spearman’s ρ = 0.214, *P* = 0.012). Similarly, pDC infiltration correlated positively with perivascular (Spearman’s ρ = 0.49, *P* = 0.000) and periadnexial lymphocytic infiltrate, abundance of mucin (Spearman’s ρ = 0.337, *P* = 0.000), neutrophilic infiltrate (Spearman’s ρ = 0.251, *P* = 0.001), and MXA scoring (Spearman’s ρ = 0.316, *P* = 0.000). MXA scoring correlated positively with vacuolar alteration of basal layer (Spearman’s ρ = 0.292, *P* = 0.000), perivascular (Spearman’s ρ = 0.378, *P* = 0.000) and periadnexial lymphocytic infiltrate (Spearman’s ρ = 0.297, *P* = 0.000), and abundance of mucin (Spearman’s ρ = 0.226, *P* = 0.007). The only negative correlation was observed between abundance of mucin and vacuolar alteration of the basal layer (Spearman’s ρ = −0.268, *P* = 0.001).

### Distribution of the lupus erythematosus subtypes within eight inflammatory groups (Interferon, B cell, plasmacytoid dendritic cell, plasmacytoid dendritic cell + interferon, interferon + B cell, B cell + plasmacytoid dendritic cell, all high, all low)

**FIGURE 2:**
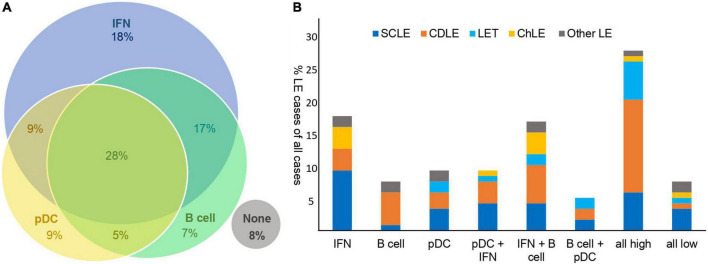
Classification of lupus erythematosus (LE) skin samples with regard to significant expression of B cell-, pDC-, and interferon-markers into eight inflammatory expression groups **(A)** and the distribution of LE subtypes within those expression groups **(B)**. **(A)** Division of the *n* = 119 LE skin samples according to their major type of immunological infiltrate assessed via staining for CD20 (B cells), CD123 (pDCs) and MXA (as IFN surrogate) into eight expression groups: IFN, B cell, pDC, pDC + IFN, IFN + B cell, B cell + pDC, all high, all low. Expression scores > 1 were considered significantly and characteristically infiltrated, and LE cases were grouped accordingly. **(B)** Distribution of different LE subtypes (SCLE, CDLE, LET, ChLE and other LE) within the expression groups. The ratio of LE subtypes is shown proportionally as % cases of all cases (*n* = 119).

Secondly, we aimed to classify LE skin samples with regard to dominating or significant expression of inflammatory markers. The majority of LE samples (59%) showed overlaps between dominating infiltrating cell types or MXA expression, while 34% displayed mutually exclusive significant expression of CD20, CD123 or MXA. *N* = 119 lupus samples were stratified according to the expression score. Samples with scores > 1 defined as significant were grouped into eight expression or infiltration groups: exclusively expressing (1) MXA (IFN-group), (2) CD20 (B cell-group) or (3) CD123 (pDC-group) (examples shown in Supplementary Figure 1); secondly, to overlapping significant expression of two of the three markers: (4) CD123 and MXA (pDC-IFN), (5) MXA and CD20 (IFN-B cell), (6) CD20 and CD123 (B cell-pDC); and thirdly, to a (7) highly infiltrated group displaying high CD20, CD123 and MXA expression scores (all high group). Group 8 (all low group) did not show either significant expression (scores ≤ 1) (Figure 2A). The majority of samples showed overlapping rather than mutually exclusive expression, with the highly infiltrated group (all high) encountering little over one fourth of all cases.

Next, we examined, whether expression groups overlapped with lupus subtype (Figure 2B). Generally, the IFN-based groups (1 and 4) encountered predominantly SCLE cases, followed by CDLE, ChLE and LET; the B cell-based groups (2 and 5) mainly displayed CDLE cases, followed by SCLE, ChLE and LET. The pDC-based groups showed a balanced distribution with a tendency toward SCLE, followed by CDLE and LET cases. The highly infiltrated group comprised mainly of CDLE cases and balanced distribution of LET and SCLE, and the poorly infiltrated group mainly of SCLE cases and a balanced distribution of SCLE, ChLE, LET and CDLE.

### Diffuse or pseudofollicular B cell infiltrates are present in different lupus subtypes and expression groups

**FIGURE 3:**
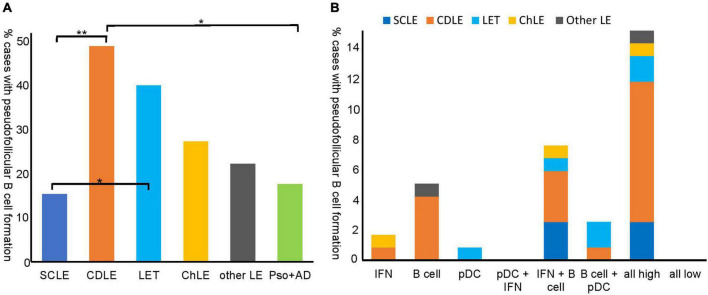
Pseudofollicular B cell formation in different lupus erythematosus (LE) subtypes and inflammatory control skin samples **(A)** and pseudofollicular B cell formation in inflammatory expression groups **(B)**. **(A)** % cases with pseudofollicular B cell formation (not overall B cell infiltration) in the LE subtypes and controls is shown proportionally to number cases of the according subtype [*n* = 39 subacute cutanous LE (SCLE), *n* = 45 chronic discoid LE (CDLE), *n* = 15 LE tumidus (LET), *n* = 11 chilblain LE (ChLE), *n* = 9 other LE and *n* = 17 inflammatory control samples (psoriasis, atopic dermatitis)]. Pseudofollicular B cell formation was defined as clustering of B cells within the inflammatory infiltrate. **(B)** % cases with pseudofollicular B cell formation proportionally to all LE cases (*n* = 119) shown with regard to inflammatory expression groups that are introduced in Figure 2.

Since B cell infiltration and function in LE has not yet been fully elucidated, we focused on the B cell based infiltration groups (groups 2, 5–7) (Figure 2B). B cell distribution showed either a diffuse (66%) or an arrangement in pseudofollicular structures (34%). Although pseudofollicular B cell formation was seen in all subtypes of LE, the occurrence varied vastly between the subtypes (Figure 3A). While 49 % of CDLE cases presented with clustered B cells, only 15% did so in SCLE, 40 % in LET and 27% ChLE (Figure 3A). B cell clusters were found significantly more often in CDLE compared to SCLE (*p* = 0.001) or inflammatory controls (*p* = 0.022), and in LET compared to SCLE (*p* = 0.011) (Figure 3A).

As a next step, we evaluated the presence of pseudofollicular B cell structures in the different expression types of LE. Interestingly, the majority of cases with pseudofollicular B cell clusters were present within the highly infiltrated subgroup (Figure 3B), and, in line with that, pseudofollicular B cell formation correlated significantly with B cells (Spearman’s ρ = 0.644, *P* = 0.000) and pDC infiltration (Spearman’s ρ = 0.243, *P* = 0.001). The second most cases were present in the IFN + B cell expression group (Figure 3B).

In a prior study our group showed that BLyS was expressed by keratinocytes in lesional LE skin, while its receptor, BAFF-R, was expressed by B cells. Therefore, we aimed to investigate whether BAFF-R was present in LE skin samples. We analyzed BAFF-R staining in *n* = 119 LE skin samples. BAFF-R expression was semi-quantitatively scored from 0: no expression; 1: scant; 2: plenty to 3: abundant expression. BAFF-R expression was mainly found in B cell clusters and, accordingly, correlated positively with presence of pseudofollicular structures (Spearman’s ρ = 461, *P* = 0.000) and with B cell counts (Spearman’s ρ = 0.397, *P* = 0.000). BAFF-R did not correlate significantly with pDCs, MXA expression, or other surrogates for inflammation. BAFF-R expression did not show a correlation toward a LE subtype.

### Fluorescent multiplex immunohistochemistry of B cell clusters in different subsets of cutaneous lupus erythematosus

To further investigate potential functions of B cells arranged in clusters in CLE samples, fluorescent multiplex-immunohistochemical analyses of histological probes were performed. The multiplex tool included four immunological markers being CD20 for B cells, BAFF-R as a B cell survival and persistence marker, MHC II for antigen-presenting cells as well as CD80/86 for T cell activation properties. For this analysis we specifically chose *n* = 17 cases of CLE with B cell clusters including five cases of SCLE, ten cases of CDLE and two cases of LET. Since we aimed for a deeper insight into the function of pseudofollicularly arranged B cells in the skin rather than giving a representative overview over LE subtypes, cases were chosen based on the infiltration seen in the conventional CD20 immunhistochemistry staining and do not represent the aforementioned subtypes. For the analysis in each probe, the total cell count was determined as well as the marker–positive cells. Additionally, proportions were determined. Analyzed cells of interest were classified as follows: CD20+ cells (B cells), CD20+ BAFF-R+ cells (BLyS-sensitive B cells), CD20+ BAFF-R+ MHCII+ cells (antigen-presenting, BLyS-sensitive B cells) and CD20+ BAFFR+ MHCII+ CD 80/86+ cells (antigen-presenting, T cell activating, BLyS-sensitive B cells).

**FIGURE 4:**
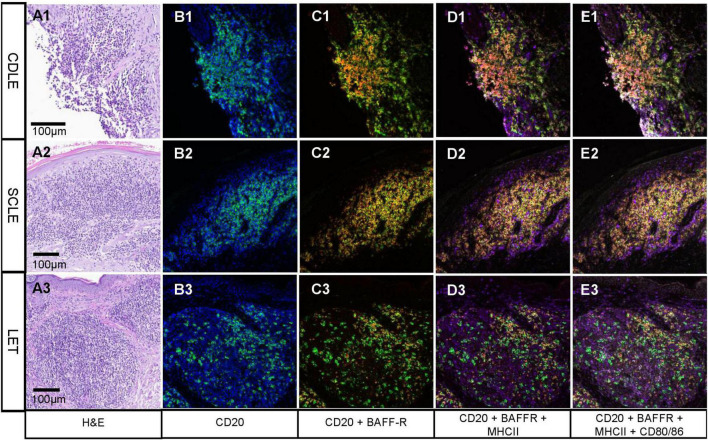
Fluorescent multiplex-immunohistochemistry focusing on B cell clusters in different subsets of cutaneous lupus erythematosus (CLE). **(A1-3)** Hematoxylin and eosin staining showing lesional inflammation in different subtypes of CLE: chronic discoid LE (CDLE) **(A1)**, subacute cutaneous LE (SCLE) **(A2)** and LE tumidus (LET) **(A3)**; scale bar 0,1 mm. B-E Immunofluorescence pictures from multiplex InSituPlex (ISP) immunohistochemistry of lesional inflammation in CLE. B1-3 cell nuclei are visualized by DAPI in blue and CD20 in green. CD20 (B cells) are localized in dense (e.g., **B1**) or scattered clusters [e.g., **(B3)**]. **(C1-3)** CD20 is visualized in green and BAFF-Receptor (BAFF-R) in red. Yellow represents CD20 and BAFF-R colocalization, which can be observed in most B cells. **(D1-3)** MHC II is visualized in purple, which is staining the B cells and other immune cell populations around them. **(E1-3)** CD80/86 is visualized in white, which colocalizes with a portion of the C20+ BAFF-R+ MHC II+ B cells. Pictures shown here were chosen based on strong expression for visualization and therefore do not represent infiltrates of the subtypes.

In each specimen, all markers were found in different ratios. In average we found 17% (1 – 41%) B cells (CD20), 13% (0 – 41%) BLyS sensitive B cells (CD20+ BAFF-R+), 12% (0 – 39%) antigen presenting B cells (CD20+ BAFF-R+ MHCII +) and 1% (0 – 3%) antigen-presenting, T cell activating, BLyS-sensitive B cells (CD20+ BAFF-R+ MHCII + CD 80/86). The exact numbers can be seen in Supplementary Table 2. As we expected, the number of B cells varied strongly between the samples depending on the size of clusters. Interestingly, each CLE subtype displayed samples with high infiltration and presented B cell formation in a similar manner, as well as cases with a scant B cell infiltration. In Figure 4, examples of prominent B cell clusters in CDLE, SCLE and LET are depicted. Again, samples were chosen based on high infiltration for visualization rather than being representative of the CLE subtype. In the H&E micrographs, areas with a dense cellular infiltrate are shown. Staining for B cells (CD20 +) shows different patterns: (1) the formation of dense clusters (B1), (2) formation of rather loose clusters (B3), single cells and diffuse infiltration (B3). In a multitude of B cells, co-localization with BAFF-R and MHC II can be observed (C1-3, D1-3), indicating an antigen-presenting role for lesional B cells. BAFF-R is almost exclusively expressed by CD20 positive cells, whereas MHC II is also expressed by other immune cells in the CLE typical infiltrate, showing different antigen-presenting cells being present. In contrast, the number of CD80/86 positive cells was rather low in all of the samples.

## Discussion

In this study we were able to show that CLE lesions present with heterogeneous infiltration patterns in addition to autoreactive cytotoxic T cell populations. Inflammatory patterns are dominated by pDCs, B cells, or a strong interferon expression. As described before, the extent and composition of the inflammatory infiltrate varies depending on CLE subtype and presence of systemic disease (5, 11, 22, 23). The most prominent difference can be observed between CDLE and SCLE, which revealed CDLE as generally stronger infiltrated especially concerning B cells and B cell cluster formation, which is in line with present literature (11). However, infiltration patterns may vary between CLE subtypes, and CLE subtype alone cannot robustly predict the infiltration pattern and the dominating cell type. The heterogeneity of inflammation in CLE might explain the clinical heterogeneity, which may be attributed to alternating underlying pathomechanisms in CLE subtypes. For example, overactivation of type 1 IFN pathway triggering cytotoxic anti-keratinocyte immune response is a central characteristic of CLE (22). In CLE, interferon is produced in large amounts either by pDCs or keratinocytes (24–28). We identified an infiltration group dominated by a strong interferon expression accompanied by high pDC infiltration, and another IFN-based group with low or absent pDC infiltration. This finding suggests that keratinocytes are the leading IFN producers in the IFN+/pDC- group of CLE patients. Of note, although both groups comprised of mainly SCLE cases, they both encountered CDLE cases as well as ChLE and others. The fact that pDC maturation is IFN-dependent in spite of their absence in this group, highlights once again the heterogeneity and complexity of CLE pathogenesis (28). Further studies are warranted investigating mechanisms in which cases pDCs are IFN-sensitive and on why not all IFN-highly expressing CLE cases are pDC infiltrated.

A limitation of our study is that we used a semi-quantitative approach. This does not necessarily reflect the total number of cells present within the specimen. In contrast, O’Brian and colleagues used a different approach semi-quantitatively stratifying according to the percentage of infiltrating cells (“0: < 1%, 1: 1–25%, 2: 26–50%, 3: > 50%”) (8). However, in our scoring system, the intensity of marker expression is taken into account, which is not applicable in other scoring systems. Secondly, we used MXA as surrogate for IFN. MXA is a protein specifically induced by type I and III interferons (29). Therefore, more specifically, it does reflect a signature of types I and III IFN. However, no information about the source of IFN- and MXA-production is given. Especially in cases that present with a parallel strong MXA expression and B cell or pDC infiltration (expression groups pDC-IFN and IFN-B cell) the underlying mechanisms of immune regulation should be further elucidated.

In our cohort, around 50% of LE skin samples were significantly B cell infiltrated. It is well known, that T and B cells bear the potential to form clusters mimicking lymphoid structures in different autoimmune diseases including cutaneous and systemic LE (30–32). Tertiary lymphoid neogenesis with a high organization level of B and T cells has been described in SLE-associated nephritis (33, 34). B cell cluster formation is present in 34% of our CLE samples, with predominance in CDLE or in the highly infiltrated expression group. While we did not show the structure of B cell clusters in detail, a priorly published small series of LEP cases also indicated the formation of tertiary lymphoid structures in LEP skin samples (35), which is in line with our proposed role for pseudofollicular B cell formation in CLE.

While mature B cells, which circulate through the blood and accumulate in the follicles of secondary lymphoid tissues, are heavily studied in detail, “skin-specific” B cells are not well described (36, 37). Skin specific B cells represent a heterogeneous population which (a) express typical skin-homing receptors, (b) present a different immune-phenotype compared to nodal B cells, (c) can activate T cells at the site of inflammation and (d) can increase local antibody production (36). Phenotypically, these B cells (CD20+), express the MHCII complex and may interact with T cells via co-stimulatory molecules like CD80/86 (38). Up until now, the definitive role for B cells in LE remains unclear. Steinmetz et al. performed intrarenal staining for MHCII in CD20-rich regions in samples of lupus-associated nephritis and proposed an antigen-presenting role (33). In this study, in a subset of lesional cells, we show direct co-expression of CD20, MHCII and CD80/CD86 via multiplex immunohistochemistry. This finding strongly indicates that B cells, especially those arranged in clusters, have antigen-presenting and T cell-activating functions. Yang et al. described a population of regulatory B cells in SLE patients (39). It is possible that different subpopulations of B cells are present in LE harboring different functions, and further research is needed, accordingly. Interestingly, we observed that the majority of lesional, clustered B cells also expressed BAFF-R. This could explain the efficacy of Belimumab in CLE despite negative serum autoantibody levels (1, 2, 11). Belimumab is currently under clinical investigation for CLE, and results are expected in the near future (EudraCT Number: 2017-003051-35). Taking into account that >50 % of CLE cases are B cell infiltrated, we expect a favorable therapeutic response, especially in patients with B cell infiltrates. In contrast, other samples presented with a strong IFN or pDC dominated infiltrate only. This subset might rather profit from pDC- or IFN-blocking agents, such as Anifrolumab.

## Conclusion

Taken together, this comprehensive study highlights the importance of histological assessment of the immunological infiltrate in LE skin lesions. Expanding the clinical classification of CLE subtypes, we suggest immunohistochemical assessment of the infiltrate for therapeutical decision making. Especially with regard to emerging targeted treatment options, predictive biomarkers are desirable for personalized therapy. Our findings should be confirmed in larger cohorts within prospective studies. Moreover, we suggest to study in detail the function of cutaneous B cell subsets in various autoimmune diseases.

## Data availability statement

The original contributions presented in this study are included in the article/Supplementary material, further inquiries can be directed to the corresponding author.

## Ethics statement

Ethical review and approval was not required for the study on human participants in accordance with the local legislation and institutional requirements. The patients/participants provided their written informed consent to participate in this study. The study was approved the Local Ethical Committee of Bonn (090/04, 153/18AMG-ff).

## Author contributions

JW conducted and supervised the study. DN was involved in the study design and concept. LV and TG conducted the statistical analyses and drafted the manuscript. LV, TG, DN, and AS scored the samples and performed the data evaluation. SB performed the experiments. All authors revised the manuscript for critical intellectual content, read and approved the final version of the manuscript.

## Supplementary material

Supplementary Figure 1 Representative examples of LE skin samples classified with regard to the three expression groups IFN, B cell and pDC. Shown are H&E **(A1-3)**, MXA **(B1-3)**, CD20 **(C1-3)** and CD123 **(E1-3)** staining of three representative samples classified into the IFN, B cell and pDC expression group. Scale bars with a scale of 100 μm are shown.
