# Supplementary material for: Characterization of B cells in lupus erythematosus skin biopsies in the context of different immune cell infiltration patterns
Source: Front Med (Lausanne). 2022 Nov 10;9:1037408. doi: 10.3389/fmed.2022.1037408 (PMC9685332; doi:10.3389/fmed.2022.1037408)
Supplement: Supplementary file 1 [file Table_1.pdf]

Diagnosis	n	Gender [%]			Age			Localization (n)			
		male	female	NA	range	mean	NA	head/ neck	trunk	extremities	NA
**LE**	**119**										
ACLE	1	0	100	0	47	47	0	1	0	0	0
SCLE	39	34	66	8	24 - 92	58	8	2	16	12	9
LET	15	17	83	3	24 - 82	58	1	1	7	2	4
CDLE	45	30	70	22	15 – 91	57	22	14	5	5	20
ChLE	11	55	45	0	21 – 78	55	0	0	1	10	0
SLE	3	33	67	0	33-76	57	0	0	1	1	1
other CLE	5	0	100	4	52	52	4	0	0	1	4
**Controls**	**17**										
AD	6	50	50	0	17 – 82	54	0	0	1	5	0
PSO	11	64	36	0	23 – 91	59	0	0	5	5	1

Supplemental Table 1. Patient characteristics. Shown are diagnosis, gender, age and localization of skin biopsy of n=119 lupus and n=17 inflammatory control patients. LE=lupus erythematosus; ACLE=acute cutaneous LE; SCLE=subacute cutaneous LE; LET=LE tumidus; CDLE=chronic cutaneous LE; ChLE=Chilblain LE; SLE=systemic LE; other CLE=other cutaneous LE; AD=atopic dermatitis; PSO=psoriasis.
