# Supplementary material for: Characterization of B cells in lupus erythematosus skin biopsies in the context of different immune cell infiltration patterns
Source: Front Med (Lausanne). 2022 Nov 10;9:1037408. doi: 10.3389/fmed.2022.1037408 (PMC9685332; doi:10.3389/fmed.2022.1037408)
Supplement: Supplementary file 2 [file Table_2.pdf]

Diagnose	All cells (n)	CD20 (n)	CD20+ MHCII+ (n)	CD20+ MHCII+ BAFF-R+ (n)	CD20+ MHCII+ BAFF-R+ CD80/86+ (n)	All cells (%)	CD20 (%)	CD20+ MHCIII (%)	CD20+ BAFF-R+ (%)	CD20+ MHCII+ BAFF-R+ (%)	CD20+ MHCII+ BAFF-R+ CD80/86+ (%)
CDLE	41068	3570	3548	2925	838	100	9	9	7		2
CDLE	60094	19361	19146	15756	1694	100	32	32	26	26	3
CDLE	13878	3489	3229	3137	40	100	25	23	24	23	0
CDLE	37592	807	650	177	0	100	2	2	0	0	0
CDLE	29763	4073	3907	1023	28	100	14	13	3	3	0
CDLE	65732	6155	5663	5167	106	100	9	9	8	8	0
CDLE	55245	15687	15503	8314	108	100	28	28	15	15	0
CDLE	68229	17174	14202	13020	1872	100	25	21	22	19	3
CDLE	17401	3013	2598	1826	457	100	17	15	11	10	3
CDLE	18086	1425	1198	1111	91	100	8	7	7	6	1
LET	79888	22792	20482	7519	237	100	29	26	10	9	0
LET	69683	6638	6638	4773	674	100	10	10	7	7	1
SCLE	23948	1500	1471	909	4	100	6	6	4	4	0
SCLE	46400	12732	12501	7522	190	100	27	27	16	16	0
SCLE	38985	1737	1643	928	523	100	4	4	2	2	1
SCLE	52636	25330	22718	20283	1035	100	48	43	41	39	2
SCLE	20359	965	965	592	27	100	5	5	3	3	0

Supplemental Material 3. Absolute and relative numbers of cells expressing CD20, MHCII, BAFF-receptor and CD80/CD86 in LE skin biopsies assessed via multiplex immunohistochemistry. Shown are absolute numbers of cells present in skin biopsies, as well as absolute and relative (%) numbers of cells expressing CD20, MHCII, BAFF-receptor (BAFF-R) and CD80/CD86 assessed via multiplex-immunohistochemistry in different LE subtypes: chronic discoid LE (CDLE), LE tumidus (LET), and subacute cutaneous LE (SCLE). Samples were chosen based on strong CD20 expression and are not necessarily representative for the respective LE subtype.
