# Supplementary figures and images for: Characterization of B cells in lupus erythematosus skin biopsies in the context of different immune cell infiltration patterns
Source: Front Med (Lausanne). 2022 Nov 10;9:1037408. doi: 10.3389/fmed.2022.1037408 (PMC9685332; doi:10.3389/fmed.2022.1037408)

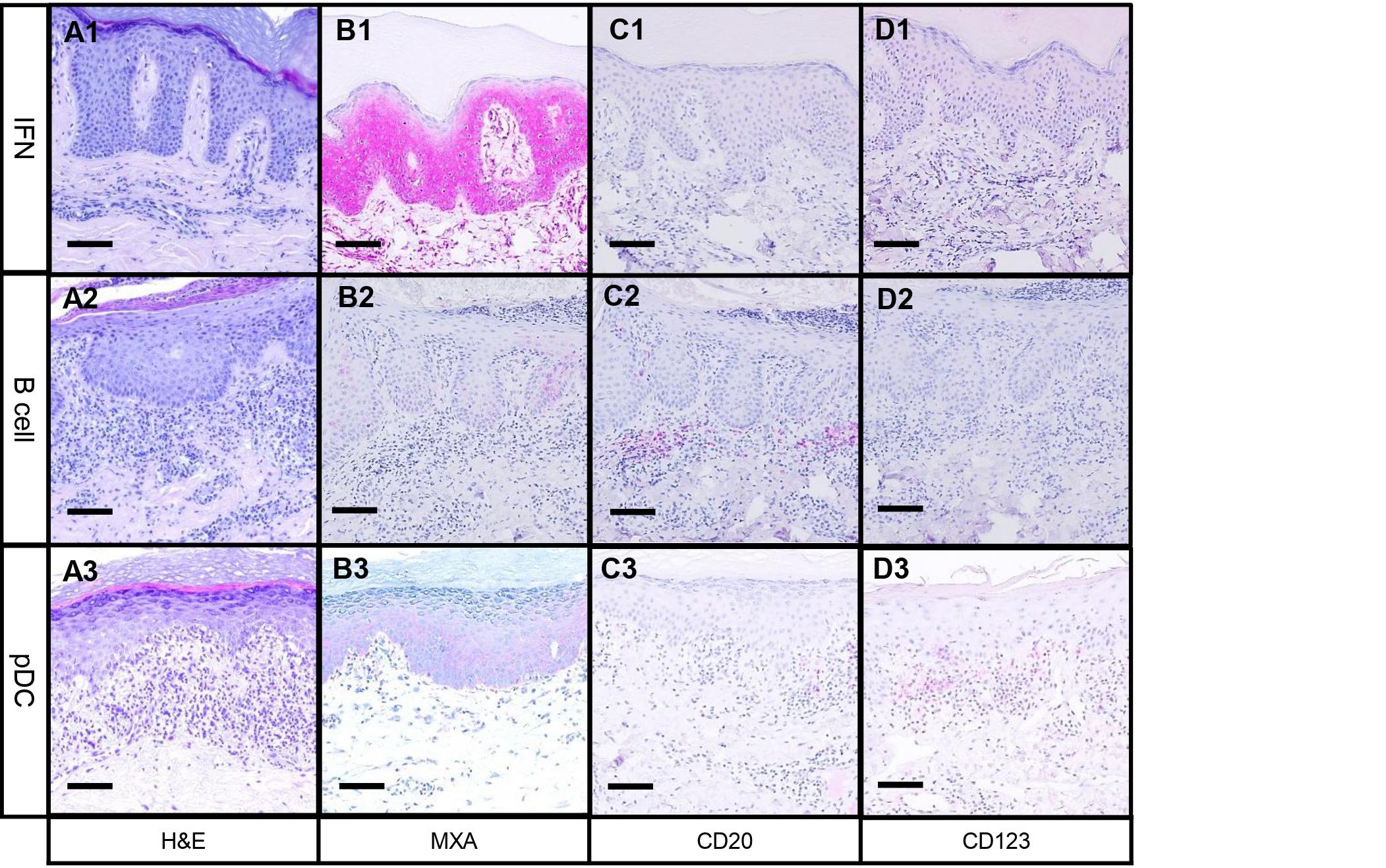

Supplement: Supplementary Figure 1 — Representative examples of LE skin samples classified with regard to the three expression groups IFN, B cell and pDC. Shown are H&E (A1-3), MXA (B1-3), CD20 (C1-3) and CD123 (E1-3) staining of three representative samples classified into the IFN, B cell and pDC expression group. Scale bars with a scale of 100 μm are shown. [file Image_1.JPEG]
